# Protocol for Moving On: a randomized controlled trial to increase outcome expectations and exercise among breast cancer survivors

**DOI:** 10.1002/nop2.119

**Published:** 2018-01-03

**Authors:** Rachel Hirschey, Gretchen Kimmick, Marilyn Hockenberry, Ryan Shaw, Wei Pan, Isaac Lipkus

**Affiliations:** ^1^ Duke University School of Nursing Durham NC USA; ^2^ Duke University School of Medicine Durham NC USA

**Keywords:** breast cancer, breast cancer survivors, cancer, nurses, nursing, protocol, RCT, survivors

## Abstract

**Aim:**

The aim of this study was to test the feasibility and fidelity of an intervention, Moving On, aimed to increase outcome expectations OEs (i.e. what one expects to obtain or avoid as a result of a behaviour) and exercise among breast cancer survivors.

**Design:**

Randomized controlled trial

**Methods:**

Intervention arm participants will be given a theory‐guided booklet that was co‐created by the research team and three physically active breast cancer survivors who exercise to manage late and long‐term treatment effects. Attention control arm participants will be given a similar booklet focused on diet. Participants will have 1 week to complete reading, writing and reflecting activities in the booklets. Study outcomes will be measured through online surveys; exercise will also be measured objectively with a Fitbit^®^. Four weeks postintervention, participants’ thoughts about the usefulness, strengths and weakness of the intervention booklet will be assessed. OEs and exercise will be measured at baseline, 4‐, 8‐ and 12‐week postintervention.

## 1 INTRODUCTION

Breast cancer is the most prevalent cancer among women in 145 countries (World Health Organization, 2011). Due to successful treatments for these patients, there are an estimated five million breast cancer survivors worldwide (World Health Organization, 2011). Unfortunately, many survivors experience late and long‐term effects, sometimes as long as 10 years after the completion of the treatment (Kenyon, Mayer, & Owens, 2014). Many of these effects may be decreased by exercise. For example, exercise has been shown to improve body image, self‐esteem, emotional well‐being, social functioning, anxiety, fatigue, sexuality (Mishra et al., 2012), pain (Irwin et al., 2015), cardiac disease risk, bone health (Kirkham, Bland, Sayyari, Campbell, & Davis, 2016) and possibly cognitive function (Campbell et al., 2017; Myers, Koleck, Sereika, Conley, & Bender, 2017). Additional exercise benefits for breast cancer survivors may include improved longevity. Specifically, associations have been observed between levels of exercise and cancer recurrence, new primary cancers, cancer‐related mortality and all‐cause mortality, with decreased rates of 28%, 21%, 33% and 46% respectively (Dieli‐Conwright, Lee, & Kiwata, 2016). To potentially achieve these exercise benefits, the American Cancer Society recommends that cancer survivors engage in a minimum of 150 weekly minutes of moderate‐intensity exercise (Rock et al., 2012). However, only from 17% (Smith & Chagpar, 2010) to 37% (Blanchard, Courneya, & Stein, 2008) of breast cancer survivors adhere to these recommendations. Interventions are needed to increase these exercise levels and potentially improve the quality and duration of breast cancer survivorship.

## 2 BACKGROUND

Interventions that focus on exercise outcome expectations (OEs) may be useful in increasing exercise levels among breast cancer survivors. OEs refer to what people expect to obtain or avoid by engaging in a behaviour (Bandura, 2004). People exercise because they believe that it will produce desired and mitigate undesired outcomes. According to several prominent health behaviour change theories, high OEs lead to behaviour change (Ajzen, 1991; Bandura, 2004; Prochaska, Redding, & Evers, 2002). Among non‐cancer populations, individuals who expect more positive and less negative outcomes of exercise have stronger intentions to exercise and tend to exercise more (Brassington, Atienza, Perczek, DiLorenzo, & King, 2002; Schutzer & Graves, 2004).

Little is known about how OEs influence exercise among breast cancer survivors. Two studies that helped increase exercise among breast cancer survivors manipulated OEs by emailing participants “realistic expectations of exercise” (Hatchett, Hallam, & Ford, 2013) and “addressing” OEs during counselling sessions. This suggests that targeting OEs is effective for this group. However, these previous interventions targeted several constructs at once; thus, the unique effects of OEs on exercise remain unknown. Furthermore, no interventions have targeted all the dimensions of OEs. Dimensions of OEs include: (i) importance—value placed on the outcome(s); (ii) certainty—perceived probability outcome(s) will occur; and (iii) accessibility—the frequency with which outcome(s) are considered (Gross, Holtz, & Miller, 1995; Olson, Roese, & Zanna, 1996; Petty & Krosnick, 2014). The first of these dimensions importance can be increased through elaboration of why outcomes are desirable, certainty can be increased by vicarious experience or the observation of another person obtaining outcomes and accessibility can be increased by implementing methods to prompt individuals to think about the association between exercise and its outcomes (Fazio, 1995; Wegener, Downing, & Krosnick, 1995). Vicarious experience can be achieved through narrative stories. Narratives are stories told in first person that connect the reader with the narrator and the narrator's experience (Bell & Bell, 2013). This connection results in the reader considering the information as more personally relevant, processing it more deeply and better retaining it (Hinyard & Kreuter, 2007). For example, as an active breast cancer survivor elaborates on outcomes personally experienced from exercise, the inactive breast cancer survivor becomes more certain that she, too, will experience similar outcomes if she exercises (Gross et al., 1995; Hinyard & Kreuter, 2007; Hopfer, 2012; Kreuter & Wray, 2003; Kreuter et al., 2008; Shaffer & Zikmund‐Fisher, 2013; Wegener et al., 1995). The physically active survivor is able to provide practical knowing (i.e. the knowledge that comes from doing what is proposed), derived from participatory action research (Heron & Reason, 1997, 2008). Previous research suggests that breast cancer survivors are influenced to act on messages received from other breast cancer survivors (Hopfer, 2012).

Breast cancer survivors often do not recognize the potential of exercise to help manage late and long‐term effects (Hirschey, Docherty, Pan, & Lipkus, 2016). They have low OEs of exercise having an impact on recurrence and mortality risk (Karvinen & Vallance, 2015). In one study, only 30% of survivors said that they thought exercise may decrease recurrence risk (Hirschey, Docherty, et al., 2016). However, survivors are typically motivated to change behaviours they believe will improve their long‐term outcomes and quality of life (O'Neill et al., 2013). Thus, increasing OEs may be effective to motivate exercise among breast cancer survivors.

**Figure 1:**
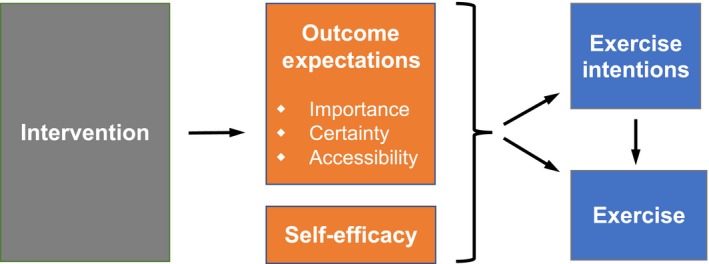
Theoretical framework

The theoretical framework guiding this study (Figure 1) is adapted from Bandura's self‐efficacy theory (Bandura, 1982); the theory proposes that exercise increases when people both expect desired outcomes will occur (i.e. have high exercises OEs) (Bandura, 2004; Hatchett et al., 2013; Rogers et al., 2009, 2013) and believe that they can perform exercise (i.e. have high exercise self‐efficacy) (Bandura, 2004; Loprinzi & Cardinal, 2013). In contrast to the many studies targeted to increase self‐efficacy, tested strategies to increase OEs are few. Therefore, this study focuses on increasing OEs. This framework is novel because multiple OE dimensions are considered, importance, certainty and accessibility. Prior interventions have simply informed participants about exercise benefits, thereby failing to address multiple OE dimensions (Hatchett et al., 2013; Rogers et al., 2009; Short, James, Girgis, D'Souza, & Plotnikoff, 2014). Conversely, this study is designed to target and increase all three OE dimensions, which in unison are hypothesized to increase exercise intentions (the most proximal predictor of behaviour) (Ajzen, 1991; Scholz, Keller, & Perren, 2009) and exercise. This approach may be more successful than previous attempts to increase exercise OEs.

Successful exercise interventions often require numerous resources such as health coaches, exercise trainers and equipment. Subsequently, many of the best interventions are not translated into practice. Therefore, the exercise intervention designed and tested here, Moving On, instead relies on the distribution of exercise booklets, a low‐cost exercise intervention that can be implemented into clinic settings. Exercise intervention booklets have been shown to be effective (Hirschey, Lipkus, et al., 2016; Short et al., 2014) and are a preferred (Stull, Snyder, & Demark‐Wahnefried, 2007) delivery mode among cancer survivors.

The purpose of this study is to test the feasibility of Moving On as an exercise intervention that can be translated into practice if effective. Moving On is a theory‐based, at home exercise intervention co‐created with physically active breast cancer survivors who use exercise to manage long‐term and late treatment effects. The primary aim of this study is to explore feasibility of Moving On among breast cancer survivors. A secondary aim is to test intervention effects on OEs and exercise. The secondary aim tests two hypotheses: first, that OE importance, certainty and accessibility will increase more in the intervention compared to the attention control arm; second, that exercise will increase more in the OE arm than the attention control arm.

## 3 THE STUDY

### 3.1 Design

This feasibility study is a randomized two‐arm trial. Eligible participants will provide written, informed consent and be given a Fitbit^®^ to wear for 2 weeks to establish baseline activity level. At baseline, they will also complete online questionnaires assessing OEs, self‐efficacy and exercise. Participants will receive an intervention or control booklet via mail and will have 1 week to complete it. Follow‐up measures will be collected at 4‐, 8‐ and 12‐week postintervention using the Fitbit^®^ and online questionnaires.

### 3.2 Method

#### 3.2.1 Participants and setting

Participants will be recruited at a tertiary cancer centre in the Southeastern United States. Inclusion criteria will include: (i) stage IA–IIB breast cancer diagnosis; (ii) 2 months–10 years status postsurgery, radiation and chemotherapy; (iii) ability to read and write English; (iv) no evidence of recurrence; (v) being inactive (self‐reported ≤150 min/wk moderate–strenuous‐intensity exercise); (vi) no contraindications to exercise based on the Physical Activity Readiness Questionnaire (PAR‐Q) (Thomas, Reading, & Shephard, 1992); (vii) approval for participation by an oncologic provider; (viii) access and ability to use a computer for completion of online measures; and (ix) possession of a smartphone for the Fitbit^®^ to be synced to.

#### 3.2.2 Sample size

Power analyses for mixed models were performed using the optimal design to estimate detectable effect sizes. The analyses revealed that the required sample sizes for detecting a small (δ = 0.20), medium (δ = 0.50) and large (δ = 0.80) effect size of change in OE importance, certainty and accessibility, with a power of 0.80, at a significance level of α = 0.05, are 944, 154 and 62 respectively. Due to the exploratory nature of this study, 60 patients will be recruited. This sample size will allow for examination of the strength and direction of intervention effects.

#### 3.2.3 Random assignment

Participants will be randomized with equal probability to the attention control or intervention arm. A blinded research assistant will orient patients to the study and facilitate data collection. To reduce performance bias among participants, the study will be introduced as being about important lifestyle information. Participants will be told that they will receive diet and exercise information and be randomly assigned to a group focused more on exercise or more on diet.

#### 3.2.4 Intervention

**Figure 2:**
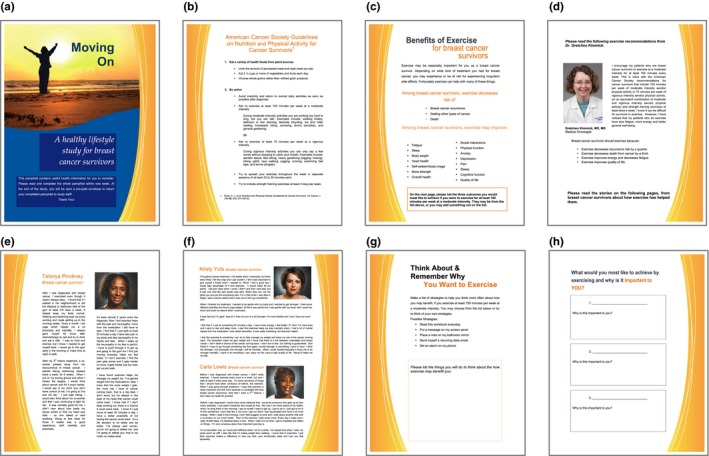
Intervention booklet note. a, cover; b and c, introduction; d, oncologist narrative/certainty Section; E and F, survivor narratives/certainty section; G, accessibility section; H, importance section

The intervention, Moving On, consists of an exercise OE booklet containing narrative messages, writing and thinking activities intended to increase OE dimensions of importance, certainty and accessibility. The booklet provides a global overview of the many and diverse positive outcomes breast cancer survivors may experience from exercise. The booklet cover (Figure 2a) contains the study name, Moving On. This name captures the stage of cancer survivorship and encourages participants to take action by moving, that is, exercising. The cover image was selected because the research team thought it was uplifting and motivational. The woman in the picture is intended to be relatable to many breast cancer survivors because she is of average to slightly above average weight, and her age and race are ambiguous. The first page of the booklet (Figure 2b) introduces the ACS recommendations on diet and exercise for cancer survivors. The second page (Figure 2c) lists outcomes associated with exercise for breast cancer survivors (e.g. decreased fatigue, improved survival). This section aims to increase awareness of the many benefits of exercise for breast cancer survivors. It explicitly states that because one has had breast cancer and treatment, these outcomes may be especially important. To increase OE importance, there is a section (Figure 2h) instructing the participant to select the three outcomes that she would most like to experience from exercise and then elaborate on all the things that will happen if those outcomes occur. OE certainty is primarily targeted in the intervention through three narrative messages, two from breast cancer survivors who exercise regularly and one from an oncologist (Figures 2d–2f). Each survivor narrative is a few long paragraphs, written in first person and includes a photograph of the author. The survivors’ narratives summarize the women's personal stories of: (i) cancer treatments and side effects they experience; and (ii) outcomes obtained as a result of exercise and how achieving these outcomes helped them manage symptoms (e.g. stress, pain). The oncologist's narrative contains: (i) her personal recommendation for breast cancer survivors to exercise; and (ii) outcomes she believes survivors may obtain, based upon current research. Finally, to increase OE accessibility, the booklet instructs participants to identify at least three strategies that could motivate them to think about outcomes they may experience if they exercise regularly (Figure 2g). Suggestions (e.g. reading booklet daily, making a computer screen saver) are provided.

#### 3.2.5 Attention control arm

Participants in the attention control arm will receive a similar booklet focused on diet instead of exercise. The diet booklet includes one oncologist and one survivor narrative, created by the research team.

#### 3.2.6 Measures

**Figure 3:**
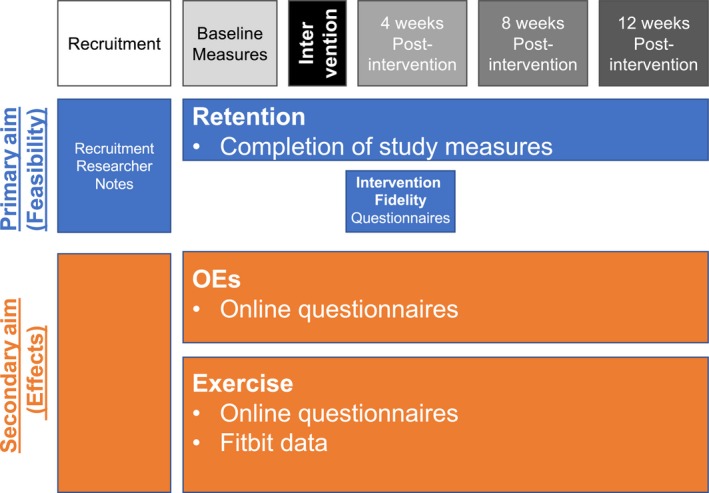
Variables and data sources to assess study aims at each measurement point

Demographic data will be collected by medical chart review and participant interview. Outcome data will be collected through researcher notes, online surveys and a waistband accelerometer, Fitbit^®^. The measurement time points, variables and data sources that will be used to address study aims are detailed in Figure 3.

##### Feasibility

Recruitment and retention of participants, use of Fitbit^®^ as an objective exercise measure, and intervention booklet fidelity will be explored to assess feasibility. The number of potential participants approached, reasons for ineligibility, or declining participation and completion of measures at each time point will be documented to assess recruitment and retention. Notes detailing communication with participants and data from Fitbit^®^ accounts will be collected to assess feasibility of using Fitbit^®^ as an objective exercise measure. To evaluate intervention fidelity, the research team constructed nine quantitative and five qualitative questions (Table 1) that were included in 4‐week postintervention measures for the intervention arm.

**Table 1:** Intervention fidelity questions included in 4‐week postintervention questionnaires

Quantitative fidelity questions (Likert scale rating 1 = not much to 5 = a great deal)
1. How much did the pamphlet make you think about how the benefits of exercise may apply to you as a cancer survivor?
2. How much did the pamphlet make you think about why the benefits of exercise are personally important for you?
3. How much did at least one of the survivor's stories resemble your own experience with breast cancer treatment and side effects?
4. How much did both of the survivors’ stories resemble your own experience with breast cancer treatment and side effects?
5. How much did at least one woman's stories make you feel that if you exercise you will experience benefits?
6. How much did the survivor's stories make you believe that you can exercise for at least 150 min per week at a moderate to strenuous intensity?
7. How much did the oncologist's story make you feel that if you exercise you will experience benefits?
8. How much did the oncologist's story make you believe that you can exercise for at least 150 min per week at a moderate to strenuous intensity?
9. How much did the pamphlet increase how often you think about the reasons you want to exercise?
Qualitative questions
1. Please write the parts of the stories that you most related to or that you found most memorable.
2. What did you do, if anything, that helped you think more often and remember your reasons to exercise?
3. What did you find most useful about the booklet?
4. What did you find least useful about the booklet?
5. Please write any additional thoughts or comments you have about this booklet.

##### Outcome Expectations

Outcome Expectations (OEs) will be measured using the multidimensional exercise OE measure for breast cancer survivors. This measure assesses the dimensions of accessibility, certainty and importance of 20 items that are possible outcomes of exercise specific to breast cancer survivors, such as decreased recurrence risk. The research team created and pilot tested this measure among a sample of 73 breast cancer survivors. The measure demonstrated excellent reliability (α 0.96–α 0.97) and stability over a 4‐week time period (*r*_*s*_ = .638–.742).

##### Exercise

Exercise intentions will be measured with Likert scale responses to three questions: (i) How motivated are you to exercise regularly over the next month? 1 = extremely unmotivated to 7 = extremely motivated; (ii) I intend to do everything I can to exercise regularly over the next month 1 = strongly disagree to 7 = strongly agree; and (iii) How committed are you to exercise regularly over the next month? 1 = extremely uncommitted to 7 = strongly committed. These questions have previously shown excellent reliability (α 0.87) in a sample of colorectal cancer survivors (Hirschey et al., 2015). Self‐reported exercise will be measured using the Godin Leisure‐Time Exercise Questionnaire (GLTEQ) (Godin, 2011). The measure demonstrates 59% specificity and 75% sensitivity among breast cancer survivors (Amireault, Godin, Lacombe, & Sabiston, 2015). Exercise will also be measured objectively with a Fitbit^®^ Flex. Fitbit^®^ has demonstrated good reliability and validity for monitoring overground energy expenditure (Adam Noah, Spierer, Gu, & Bronner, 2013). For step count outputs compared to research observer counts, concordance = 0.97–1.00 and interdevice reliability of the step count at all walking speeds = ICC ≥0.95 (Takacs et al., 2013).

### 3.3 Analysis

Baseline demographic variables will be compared between the intervention and control arms using *t*‐tests for continuous variables and Chi‐squared tests for categorical variables. Statistically significant differences will be controlled for in all analyses.

#### 3.3.1 Feasibility and fidelity

Descriptive statistics will be conducted to assess participant recruitment and retention at each time point. Recruitment will be considered feasible if 60 participants are recruited within 6 months, which is an average of one to two participants per week. This target enrolment rate is comparable to previous recruitment rates of breast cancer survivors, to other physical activity interventions, using similar recruitment strategies. For example, in one study, it took 12 months to recruit 40 participants at clinic follow‐up visits (Fields, Richardson, Hopkinson, & Fenlon, 2016), and in another study, it took 23 months to recruit 210 participants through the mail (Befort et al., 2014). Retention will be considered feasible if attrition is less than 17%, which is comparable to other home‐based exercise interventions for breast cancer survivors in which attrition ranged from 13% to 20% (Lahart, Metsios, Nevill, Kitas, & Carmichael, 2016; Pinto, Papandonatos, & Goldstein, 2013; Pinto, Rabin, Abdow, & Papandonatos, 2008; Rabin, Pinto, Dunsiger, Nash, & Trask, 2009).

Fitbit will be considered a feasible objective exercise measure if the percentage of Fitbit data obtained is equal or greater than the percentage of subjective data obtained from online questionnaires. Notes detailing Fitbit^®^‐related interactions between the research team and participants will be reviewed to identify common themes about Fitbit^®^ set‐up and use. Means and standard deviations will be calculated for the quantitative fidelity questions. Qualitative fidelity data will be examined to identify reasons for low scores (defined a priori as ≤2.0 on the five‐point Likert Scale). Common themes that inform the extent to which participants understood, completed and found the intervention booklet useful will be identified.

#### 3.3.2 Intervention effects

Two‐level multilevel modelling will be done using Proc Mixed with SAS software version 9.4. In the level‐1 model, outcomes will be modelled as a linear function of time (baseline, 4‐, 8‐ and 12‐weeks postintervention) to create growth trends. In the level‐2 model, the growth trends will be modelled as a linear function of arm (intervention vs. control). Models will be constructed for OEs, exercise intentions, subjective exercise and objective exercise. For OEs, each dimension will be modelled individually to determine if intervention effects differed between OE dimensions and the average of all dimensions will also be modelled. The level of significance will be set at 0.05, two‐tailed. Effect sizes will be calculated by dividing each beta coefficient by the square route or residual error variance for each outcome. This value is interpreted similar to Cohen's d in which a small effect is 0.2, a medium effect is 0.5 and a large effect is 0.8 (Cohen, 1988).

### 3.4 Ethics

This study was approved by the Medical Centre's Cancer Protocol Committee and Internal Review Board (Protocol #00059469).

## 4 DISCUSSION

This protocol article details the components of Moving On and how it will be tested to determine feasibility and intervention effects. There are several strengths to this study. First, Moving On is guided by a theoretical framework that is centred around a significant predictor of behaviour change, OEs (Bandura, 2004). Theory‐guided exercise interventions are usually more effective than those not guided by theory (Bluethmann, Vernon, Gabriel, Murphy, & Bartholomew, 2015). Additionally, when interventions are guided by theory, the constructs can be measured and analyses conducted to understand how the intervention operates and impacts each construct. This information aids researchers in designing more effective future interventions. Another strength of this intervention is that the population, whom it is designed to benefit (i.e. breast cancer survivors), participated in the creation of the study. The experiential knowledge these women bring to the study strengthens the likelihood that study participants will retain information about exercise OEs contained in the Moving On booklet (Bell & Bell, 2013). Finally, Moving On is an intervention that does not require expensive equipment or additional staffing such as exercise trainers or nurses to provide motivational interviewing. If Moving On is effective, it can easily be distributed during a regular follow‐up clinic visit; thus, this study has broad dissemination potential.

The planned feasibility trial of Moving On will allow the research team to test study procedures including recruitment and the use of Fitbit^®^ and online measures for data collection. The trial will also elicit feedback about what participants do and do not find beneficial about the Moving On booklet. The strength and direction of intervention effects on OEs and exercise will be identified. This information will facilitate refinement of Moving On materials and procedures in preparation for a future larger study.

Regardless of the outcomes of Moving On, information will be gained about what breast cancer survivors do with printed materials that they receive through mail or during clinic visits. Patients are commonly provided written information; however, the extent to which they read and find the information useful is rarely assessed. Due to the continuously increasing demands of breast cancer survivorship care (I. Chopra & Chopra, 2014), it is necessary to test low‐cost, simple methods of disseminating information to patients.

### 4.1 Limitations

Several limitations of Moving On must be considered. First, while a strength of the study is that the intervention requires minimal resources, some people may need a more intensive intervention. To increase exercise for some people, intensive approaches such as those that include social support or coaching may be needed. Additionally, while the theoretical framework focuses on two of the most significant predictors of behaviour, many other significant constructs such as barriers are not included. This may result in smaller effects on exercise and will also prevent analysis to control for potential confounders beyond self‐efficacy. Another important consideration is the correlation between diet and exercise behaviour change. Often when a person improves dietary practices, they also improve physical activity levels (Psouni, Chasandra, & Theodorakis, 2016). As the attention control group will receive dietary information, they may improve their dietary practices. This could in turn also lead to unintended increases in physical activity and decrease detection of intervention effects. Finally, selection bias may impact study results because people who have positive attitudes towards exercise and diet may be more likely to enrol in this healthy lifestyle study. These people may have greater motivation to exercise and be more sensitive to the intervention.

## 5 CONCLUSION

Moving On is an exercise intervention that if effective will increase levels of exercise among breast cancer survivors to ultimately improve the duration and quality of survivorship. Additionally, insights will be gained about how patients use printed materials they are given. This knowledge will inform both how to make effective behavioural interventions and how to provide important clinical information to breast cancer survivors in time‐ and cost‐effective ways as the demands of survivorship care continue to increase.
